# Incidence, risk factors, and outcomes of hospital-acquired infections with common respiratory viruses

**DOI:** 10.1017/ash.2023.367

**Published:** 2023-09-29

**Authors:** Joshua Petrie, Riley Moore, Adam Lauring, Keith Kaye

## Abstract

**Background:** We estimated the incidence of hospital-acquired respiratory virus infections (HARVIs) by viral species, and we identified risk factors for and outcomes of HARVIs. **Methods:** We identified a cohort of all inpatient admissions of ≥24 hours duration to University of Michigan hospitals during 3 study years (2017–2018, 2018–2019, and 2019–2020). HARVIs were defined as initial respiratory virus detection (adenovirus, coronaviruses, human metapneumovirus, influenza A and B, parainfluenza viruses, respiratory syncytial virus, or rhinovirus-enterovirus) in a clinical test ordered after the 95th percentile of the virus-specific incubation period. Incidence was calculated as the number of HARVIs per 10,000 patient admission days. Patient demographic and clinical characteristics were assessed as risk factors for HARVI in Cox proportional hazards models of the competing outcomes of HARVIs and hospital discharge. The association between time-varying HARVI status and the competing outcomes of discharge and in-hospital death was estimated in covariate-adjusted Cox-proportional hazards models. All analyses were performed separately for adult patients (aged ≥18 years) and pediatric patients (aged <18 years). **Results:** The overall incidences of HARVI were 8.5 and 3.0 per 10,000 admission days for pediatric and adult patients, respectively. Rhinovirus was the most common HARVI in both pediatric and adult patients, with incidences of 5.1 and 1.1 infections per 10,000 admission days, respectively. With the exception of influenza A, the incidence of HARVI was higher in pediatric patients than adult patients for all viral species. For adults, congestive heart failure, renal disease, and cancer all increased HARVI risk independent of their associations with extended hospital stays. Risk of HARVI was also elevated for patients admitted September through June relative to July admissions. For pediatric patients, chronic cardiovascular and respiratory conditions, cancer, medical-device dependence, and December admission increased risk of HARVI. Age, sex, and race were not associated with risk of HARVI for children or adults. Inpatient lengths of stay were longer for adults with HARVI compared to those without (range of virus-specific hazard ratios, 0.48– 0.77). However, estimated effects were not statistically significant for human metapneuomovirus, parainfluenza, or adenovirus. Only influenza A was associated with an increased risk of in-hospital death within 30 days of infection for adults. No HARVIs were associated with increased length of stay or risk of death for pediatric patients. **Conclusions:** The incidence of HARVI varied by viral species and was higher among pediatric patients. HARVIs increased the length of hospital stays for adults but not for pediatric patients.

**Disclosures:** None

